# What do adult outpatients included in clinical trials know about the investigational drugs being assessed: A cross-sectional study in France

**DOI:** 10.1371/journal.pone.0220383

**Published:** 2019-08-13

**Authors:** Clémentine Fronteau, Maxime Paré, Philippe Benoit, Sophie Tollec, Catherine Hamon, Vérane Schwiertz, Christian Maillard, Amélie Cransac, Christelle Volteau, Jean-François Huon, Véronique Burgeot, Martine Tching-Sin, Corinne Guérin, Laurent Flet

**Affiliations:** 1 Department of Pharmacy, Nantes University Hospital, Nantes, France; 2 Department of Pharmacy, Reims University Hospital, Reims, France; 3 Department of Pharmacy, Orléans Regional Hospital, Orléans, France; 4 Department of Pharmacy, Rennes University Hospital, Rennes, France; 5 Department of Pharmacy, Hospices Civils de Lyon, Lyon, France; 6 Department of Pharmacy, Nîmes University Hospital, Nîmes, France; 7 Department of Pharmacy, Dijon University Hospital and LNC-UMR1231, Dijon, France; 8 Clinical Research Unit, Nantes University Hospital, Nantes, France; 9 EA3826 Laboratory, Nantes University; UFR des Sciences Pharmaceutiques, Nantes University, Nantes, France; 10 Department of Pharmacy, AP-HP Cochin Hospital, Paris, France; Royal College of Surgeons in Ireland, IRELAND

## Abstract

This study aimed to assess patient investigational medication knowledge and to identify factors associated with medication understanding by adult outpatients included in clinical trials. A cross-sectional prospectively designed survey was conducted on consecutive volunteers at 21 university teaching hospitals (in France) from February to December 2014. Investigational medication understanding was assessed at the time of the first dispensing using a structured interviewer-administered questionnaire based on information obtained from the literature that provided an 8-point score. Demographic and other baseline data were collected using structured interviews. Of the 236 participants, 139 (58.9%) of the respondents were male, and the median age was 54.9 years (range: 18–83 years). The mean understanding score was 6.24 and 72.5% of the patients had a score of 6 or higher. In univariate analysis, the medication understanding score was negatively correlated with age (r = -0.15, p = 0.0247) and positively correlated with the level of education (r = 0.25, p = 0.0002). In multivariate analysis, prognostic factors of a higher medication understanding score were: graduation from high school or a higher level of education; HIV infection; phase II/III/IV studies; mention of the drug on the prescription form, and the dispensing of a single investigational medication. Only a quarter of the adult outpatients included in clinical trials had a maximum possible investigational medication understanding score. Being old and having a low level of education were found to be important risk factors for inadequate medication understanding. This and other data suggest that sponsors should encourage initiatives aimed at improving investigational medication understanding in adults enrolled in clinical trials.

## Introduction

The complexity of medication management is increased considerably when patients participate in clinical trials. Moreover, as the results of clinical trials depend on the extent of the participants’ compliance with the study requirements, the subject’s level of understanding of the medication being tested is key to ensuring that the trial is safe, that the clinical investigation is conducted properly, and that the data are reliable. In this context, medication adherence is a major issue in clinical trials conducted in an ambulatory setting [1], and it is influenced by many factors related to the patients, the investigators, and the study procedures including those related to investigational medication products (IMPs).

In compliance with the good clinical practices and with French regulatory requirements, participants newly included in a clinical trial receive information about their medications in writing: an informed consent form, a prescription form, the medication label and, sometimes, sponsor-specific documents. For the majority of patients, consent forms are often too complex to be properly understood [2–4]. This can add complexity and it can overload patients with information, thereby potentially exacerbating the level of anxiety felt by the participants. A systematic review in 2013 reported that different types of interventions aimed at improving patient knowledge of research consent forms had variable impacts [5]. Participants in a clinical trial also receive oral information from the investigators [6], and from the persons in charge of the IMP dispensation. Thus, before starting their treatment, all of the information available to the participants about their new experimental drugs is obtained from these sources. This, however, amounts to only a fraction of the information that they need during the short time they have to make a decision whether or not to participate in the trial in order to understand what is involved.

Health literacy and interventions to improve the informed consent process for patients as well as to reduce misunderstanding of the research [7] are well-documented, but an evaluation of determinants of patient understanding of medications is lacking, especially in this specific population.

The aim of the present survey was to assess patient medication knowledge (quantitative rates) in a population of adults who were included in clinical trials. A secondary outcome was to identify the factors associated with medication understanding.

## Materials and methods

COMQUEST (COMprehension QUESTionnaire) was a cross-sectional, multicenter study carried out at 21 public University teaching hospitals throughout France. To be eligible, participants had to be ≥ 18 years of age and patients or healthy volunteers included in any clinical trial in an ambulatory setting. They were to receive self-administered experimental drugs that they had not been treated with previously that were related to the clinical trial in which they had recently been included. There were no exclusion criteria. Indeed, we considered that once a participant was included in a clinical trial and the investigator had assessed their ability to understand their medication, they could participate in our survey.

Because we deliberately decided to focus on issues that relate strictly to medications, a structured medication knowledge questionnaire was specifically designed for this study. It was based on the Medication Understanding Questionnaire [8] and on the Patient’s Knowledge of their Medicines [9]. It consisted of eight questions (six from these two questionnaires and two additional questions) that focused on the therapeutic indication, the name of the medicinal product, the pharmaceutical form, the route of administration, the frequency of intake, the daily dose, the duration of treatment, and the storage conditions. Content validity was established by consultation with relevant experts (e.g., pharmacists at Nantes University Hospital). The open-ended questions were provided during face-to-face interviews and observations carried out with the participants. For each question, their answers related to a specific drug were then coded as either 0 (false) or 1 (true). As some patients might receive one or more drugs, the individual scores for each item were added and divided by the number of medications taken to yield a final averaged Medication Understanding Score (MUS) that ranged from 0 to 8 for each patient.

We also collected the participant’s socio-demographic characteristics and data related to the specific clinical trials for which they had volunteered. This included, among other things, questions relating to the following topics: the sponsorship, the clinical trial phase, the investigator’s specialty, blinding, the number of medications dispensed, IMP packaging and labeling, and factors related to the prescription form (e.g., the prescription type and the name of the drug on the prescription).

The population comprised consecutive patients who relied on hospital pharmacies for their IMP dispensing. They were prospectively invited to participate, and they were provided written information about the survey. The patients were enrolled once compliance with the inclusion criteria had been verified and verbal consent had been obtained. The IMPs were then dispensed in accordance with the protocol requirements and with a copy of the prescription form.

The questionnaire was administered in French. All of the respondents were asked exactly the same questions and in the same order. They could use the following relevant materials to answer the questions: previous oral information, the prescription, the IMP label, and any of the sponsor documents, if applicable. All of the interviewers were pharmacists involved in IMP dispensation. They were trained in use of the questionnaire and how to conduct the interviews. During the interviews, they noted any specific medication-related activities such as reading of the IMP labels or looking at the prescription and at other information sources used by patients when they answered the questions. After having completed the questionnaire session, the interviewers could provide the participants with advice according to their specific needs. This, however, was not assessed in this survey.

All of the personal data were treated confidentially and rendered fully anonymous. SAS version 9.4 software (SAS Institute Inc., Cary, NC, USA) was used to analyze the data. The patients who met all of the inclusion criteria constituted the fully eligible cohort and were hence enrolled in the study.

Demographic and other baseline data (including clinical trial characteristics) were described. Continuous data were expressed as the mean and standard deviation or the median and interquartile range when the normality assumption was not assessed. Categorical data were presented as numbers and percentages. Spearman correlation coefficients were determined for the analysis of the correlation with the age, the level of education, and the medication comprehension score of the patients. Univariate analyses were carried out to analyze associations with the score based on the Student’s t-test or Wilcoxon test. Factors associated with the medical comprehension score were secondarily analyzed with a multivariate linear regression model with a backward selection procedure. The initial model included all of the variables with a p-value < 0.05 in the univariate analysis, and the variable "Number of IMPs dispensed" as the outcome of interest. All of the statistical tests were two-sided. A p-value less than 0.05 was considered statistically significant.

COMQUEST was conducted in accordance with French law for non-interventional research. The protocol was approved by the Groupe Nantais d’Ethique dans le Domaine de la Santé (GNEDS) Ethics Committee, Nantes, France (May 13, 2014).

## Results

### Baseline characteristics of the enrolled study participants

From February to December 2014, a total of 236 volunteers were enrolled and data for all of them were included in the analysis. One person refused to take part in the survey. None of the patients failed to complete the entire interview.

The baseline demographics and characteristics of the subjects included in the survey are summarized in Table 1. The median age was 54.9 years (IQR 41.7, 65.4) (range: 18–83 years), and 58.9% were male. More than half (58.2%) had graduated from high school or achieved a higher level of education. All of them were francophones. Out of the entire sample (n = 236), 77.5% (n = 183) reported initiating an experimental treatment for the first time. Patients represented 97.9% (n = 231) of the participants while healthy volunteers accounted for 2.1% (n = 5). The participants were included in 80 different clinical trials. Infectious disease patients accounted for 29.4% (n = 68), and individuals with hematological malignancies represented 22.5% of the included participants (n = 52). They tended to be included in clinical trials sponsored by pharmaceutical companies (57.2%; n = 135), and in phase III studies (57.0%; n = 134). They participated equally in open-label and double-blind studies (45.3%; n = 107 and 45.7%; n = 108, respectively). Overall, 262 different prescribed investigational medications were dispensed to the participants, with an average of 1.1 (range 1–3 ± 0.34 SD). Most of them were administered orally. Ninety-two percent of the IMPs had a specific clinical trial packaging (n = 217) and all of them had specific labeling (as a front-of-pack label or booklet label). All of the IMPs were delivered with a prescription form.

**Table 1:** Subject and clinical trial characteristics (n = 236).

Participant characteristics		Value
Age (years)	Range	[18.4;83.3]
	Mean (± SD)	53.2 ± 15.7
	Median [IQR 25%-75%]	54.9 [41.7–65.4]
Gender	Women	97 (41.1)
	Men	139 (58.9)
Highest level of education^a^	5-year university degree or more	19 (8.6)
	4-year university degree	13 (5.9)
	2-year university degree	49 (22.3)
	High school degree	47 (21.4)
	Initial professional diploma	54 (24.5)
	No qualification	38 (17.3)
Previous participation in a clinical trial	Yes	53 (22.5)
	No	183 (77.5)
Profile of the participants	Patients	231 (97.9)
	Healthy volunteers	5 (2.1)
Patient’s disease^b^	Infectious diseases	68 (29.4)
	Hematology-oncology	52 (22.5)
	Neurology	24 (10.4)
	Gastroenterology and Hepatology	23 (10.0)
	Others	64 (27.7)
Clinical trial characteristics		Value
Sponsorship	Pharmaceutical company	135 (57.2)
	Other sponsors (hospital, health institution, or medical association)	101 (42.8)
Clinical trial phase^c^	I	7 (3.0)
	II	52 (22.1)
	III	134 (57.0)
	IV	42 (17.9)
Blinding	Open-label	107 (45.3)
	Simple-blinded	21 (8.9)
	Double-blinded	108 (45.8)
Duration of the treatment^d^	< 1 week	3 (1.3)
	> 1 week	230 (98.7)
Number of IMPs dispensed	Range	[1.0;3.0]
	Mean (± SD)	1.1 ± 0.34
	Median [IQR 25%-75%]	1.0 [1.0;1.0]
	1	212 (89.8)
	2	22 (9.3)
	3	2 (0.8)
IMP route and form	Oral–tablet or capsule	206 (87.3)
	Oral–powder	15 (6.4)
	Oral–liquid medication	7 (3.0)
	Subcutaneous–pre-filled syringe	5 (2.1)
	Ocular–eye drops	2 (0.8)
	Topical—bandage	1 (0.4)
IMP packaging	Clinical trial packaging	217 (92.0)
	Commercial packaging	19 (8.0)
IMP labelling type on packaging^c^	Front-of-pack label	113 (48.1)
	Booklet label	122 (51.9)
IMP label in French	Yes	109 (46.2)
	No	127 (53.8)
Label as a source of information^c^	Yes	96 (40.9)
	No	139 (59.1)
Clinical trial prescription form type	Specific	179 (75.8)
	Non-specific	57 (24.2)
Prescription form as a source of information^b^	Yes	95 (40.4)
	No	140 (59.6)
Drug name on prescription^e^	Yes	187 (82.4)
	No	40 (17.6)

The data are presented as the mean ± standard deviation, the median and interquartile range, or numbers with percentages in parentheses.

^a^ Missing data: 16

^b^ Missing data: 5

^c^ Missing data: 1

^d^ Missing data: 3

^e^ Missing data: 9

### Medication understanding score

Table 2 presents each item of the score and the proportion of the participants who responded correctly, while Table 3 presents the results for the overall medication understanding score.

**Table 2:** Medication understanding score (MUS) and components (Q: question) for 236 participants.

	Mean (± SD)	Volunteers with the correct answer (%)
MUS *	6.24 (1.55)	/
Q1: therapeutic indication	0.94 (0.24)	221 (93.6)
Q2: medication name	0.56 (0.49)	130 (55.1)
Q3: pharmaceutical form	0.86 (0.35)	201 (85.2)
Q4: route of administration	0.94 (0.24)	220 (93.2)
Q5: frequency	0.84 (0.36)	198 (83.9)
Q6: daily dose	0.80 (0.40)	187 (79.2)
Q7: duration of treatment	0.80 (0.40)	189 (80.1)
Q8: storage conditions	0.54 (0.50)	128 (54.2)

* the score could range from 0 to 8 for each volunteer

**Table 3:** Distribution of the participants’ overall medication understanding scores (MUS).

MUS	Participants (%)
[0–1]	0 (0.00)
[1–2]	3 (1.27)
[2–3]	2 (0.85)
[3–4]	11 (4.66)
[4–5]	12 (5.08)
[5–6]	37 (15.68)
[6–7]	61 (25.85)
[7–8]	49 (20.76)
8	61 (25.85)
Total number of participants	236

The mean score was 6.24, with a 95% confidence interval of [6.04; 6.44], and 72.5% of the patients had an understanding score of 6 or higher. A score of less than 8 occurred for 74.1% of the participants. Five questions (numbers 1; 3 to 5, and 7) had correct response rates that exceeded 80%.

Correlations between the medication understanding score and the baseline characteristics were analyzed. In univariate analysis, the medication understanding score was negatively correlated with age (years) (r = -0.15, p = 0.0247) and positively correlated with the level of education (levels as described for Table 1) (r = 0.25, p = 0.0002). No other covariate was consistently associated with the medication understanding score.

Moreover, individuals < 65 years of age; patients who had completed high school or a higher level of education; volunteers participating in studies that were not sponsored by pharmaceutical companies; phases II, III, and IV participants; volunteers included in blinded studies; HIV patients; cases where the IMP label was in French; patients who had read the IMP label, and cases where the drug’s name was mentioned on the prescription had a statistically significant higher medication understanding score (Table 4). The score did not differ based in terms of: gender; previous participation in a clinical trial; the profile of the participants (i.e., patients vs. healthy volunteers); the number of IMPs dispensed; the IMP packaging; the IMP labeling; the type of prescription form, and whether the patient had read the prescription.

**Table 4:** Factors associated with medication understanding.

Characteristics		n	MUS mean (SD)	p-value
Age	< 65years	170	6.39 (1.32)	0.0389
	≥ 65years	62	5.81 (2.03)	
Gender	Female	97	6.16 (1.63)	0.5504
	Male	139	6.29 (1.50)	
Level of education	≥ High school diploma	128	6.57 (1.35)	0.0003
	< High school diploma	92	5.78 (1.71)	
Previous participation in a clinical trial	No	183	6.15 (1.54)	0.0681
	Yes	53	6.53 (1.55)	
Profile of the participant	Patient	231	6.22 (1.55)	0.2571
	Healthy volunteer	5	7.00 (1.22)	
Patient’s disease	HIV	49	7.45 (0.87)	< 0.0001
	Other diseases	181	5.91 (1.56)	
Sponsorship	Pharmaceutical company	135	6.00 (1.61)	0.0062
	Other sponsors	101	6.55 (1.41)	
Clinical trial phase	I	7	4.50 (2.25)	0.0181
	II, III, and IV	228	6.31 (1.47)	
Blinding	Blinded studies	129	6.52 (1.53)	0.0023
	Open-label studies	107	5.90 (1.51)	
Number of IMPs dispensed	1	212	6.28 (1.51)	0.3301
	> 1	24	5.87 (1.83)	
IMP packaging	Clinical trial packaging	217	6.27 (1.53)	0.3553
	Commercial packaging	19	5.84 (1.74)	
IMP labeling type	Booklet label	122	6.09 (1.42)	0.1396
	Front-of-pack label	113	6.39 (1.68)	
IMP labels in French	No	127	5.98 (1.54)	0.0025
	Yes	109	6.53 (1.51)	
Label as a source of information	No	139	6.06 (1.50)	0.0355
	Yes	96	6.49 (1.60)	
Clinical trial prescription form type	Non-specific of the CT	57	5.96 (1.51)	0.0975
	Specific of the CT	179	6.32 (1.55)	
Prescription form as a source of information	No	140	6.09 (1.50)	0.0902
	Yes	95	6.44 (1.60)	
Drug name on the prescription	No	40	5.70 (1.45)	0.0083
	Yes	187	6.31 (1.56)	

MUS = medication understanding score; IMPs = investigational medicinal products; CT = clinical trial

We conducted additional multivariate analyses (Table 5). The initial model of multiple linear regression included: the participant’s age; level of education (i.e., whether they had graduated from high school); their disease; the sponsorship; the clinical trial phase; blinding; the IMP label language; whether they had read the IMP label; whether the drug’s name was mentioned on the prescription, and the number of IMPs dispensed. The prognostic factors identified through backward modeling were as follows: HIV infection; phase II/III/IV studies; dispensing of a single IMP; mention of the drug on the prescription form; and having completed high school or a higher level of education (Table 5).

**Table 5:** Factors associated with medication understanding (multiple linear regression; n = 206).

Variable	Estimate	SD	95% CI	p-value
Patient’s disease: HIV (vs. others)	1.49	0.23	[1.04; 1.95]	< 0.0001
Clinical trial phase: II/III/IV (vs. I)	1.27	0.53	[0.23; 2.31]	0.0169
Number of IMPs dispensed = 1 (*vs*. > 1)	0.71	0.30	[0.12; 1.30]	0.0182
Drug name on the prescription: yes (vs. no)	0.65	0.25	[0.17; 1.14]	0.0086
≥ High school diploma (*vs*. < High school diploma)	0.55	0.19	[0.17; 0.93]	0.0050

IMPs = investigational medicinal products

## Discussion

Patient understanding has been widely assessed in various fields of healthcare. We specifically evaluated the knowledge of a broad range of investigational medications used in clinical trials from various fields of research, involving diverse populations and all phases of clinical development. Our survey allowed us to assess for the first time to what extent people who volunteer for clinical research understand their experimental treatment. This survey involved patients and healthy volunteers included in clinical trials that were performed at University Hospitals. As initiating a new medication treatment is a critical period, we decided to assess the participants’ level of understanding at the time of the first dispensation of medication because this is the first time that they encounter their new experimental drug before they return home. We did not reveal the aim of the study to the investigators and their team to avoid the possibility that they might consciously or unconsciously change their practice and provide the participants with additional information. We used an eight-component medication understanding questionnaire. Although it was developed specifically for this survey, it was based on two existing tools. It provided a Medication Understanding Score (MUS) that ranged from 0 to 8. It proved to be easy to administer by the medical interviewers and it was well-understood by the participants.

Our data highlight several key points. Only 25.8% of the participants achieved the highest possible medication understanding score of 8/8. The questionnaire items that typically yielded the highest values were in regard to: the therapeutic indication, the pharmaceutical form, the route of administration, the frequency, the daily dose, and the duration of the treatment, while items regarding the medication name and storage conditions generally had the lowest scores.

In terms of patient characteristics, we found that the participant’s age and their level of education correlated with the Medication Understanding Score. Participants who were older and who had a lower level of education have less of an understanding of the investigational products. We also demonstrate that people with HIV had a significantly higher level of understanding, even after adjusting for age and the level of education. This could be explained by the HIV-specific culture of joint mobilization (multidisciplinary and patient-centered care in the context of a chronic illness) [10]. On the other hand, previous participation in a clinical trial was not found to affect the comprehension score. Thus, we were able to establish a profile for patients who are at risk of misunderstanding.

Our results also suggest that the clinical trial characteristics could affect patient comprehension: participation in a study sponsored by a pharmaceutical company (p = 0.0062), in open-label study (p = 0.0023) or in a phase I study (p = 0.0181) was associated with a statistically significant decrease in the Medication Understanding Score. Scores were also lower when the IMP labels were not in the patient’s own language. Moreover, for 40 patients the drug name on the prescription differed from that on the container label, and for nine of them, there was no drug name on the prescription. Surprisingly, the number of IMPs dispensed did not significantly affect the MUS. However, a greater number of IMPs was predictive of less understanding of the frequency and the daily dose of the treatment, as described in the field of medication adherence [11].

Part of the novelty of this survey was that it also asked the interviewers to make structured observations about the behavior of the respondents. This allowed it to be shown that almost 60% of the patients did not read the IMP labels or the prescription forms despite the fact that they were allowed access to these materials. This is a major concern for the trials’ safety, because labels and prescriptions contain useful information for patients in regard to their experimental treatment, such as the drug strength, the number of units to be taken at any time, the dosing frequency, the duration of the treatment, and the storage requirements. We found that the participants were more likely to have understood their medication when the drug name was indicated on the prescription (p = 0.0083) and when they referred to the container label (p = 0.0355) to answer the questions even though the packaging (specific to clinical trials versus authorized medications packaging) and the labeling (standard front-of-pack clinical labels versus booklet clinical labels) did not influence the comprehension score. In addition, the presence of at least two of the following three items on the container label significantly improved the participants' understanding (p = 0.0001): the name of the drug, its strength, and the route of administration.

To our knowledge, no studies to date have investigated patient comprehension of their IMPs. In many studies, the level of education and the age of the participants were found to significantly affect other aspects of their understanding [12–15] or adherence [16]. In order to improve participant investigational medication understanding, sponsors of clinical trials and investigators should consider providing training sessions by targeting patients at risk of misunderstanding, for less educated and for older people in particular. This survey also showed that they should consider the volunteers included in studies sponsored by a pharmaceutical company and in phase I studies. Similarly, the needs of patients included in open-label trials should also be assessed. The people in charge of IMP dispensing should also be aware of these results so as to target their patient-centered interventions.

Based on the findings of this survey, sponsors could offer proper tools to trial sites to gain insight into patient medication understanding. The complexity of participating in a clinical trial could be offset by improving medication materials. Firstly, there is a need to improve the design and the content of clinical labels. IMP container labels are often printed in small fonts that are difficult to read. Aside from the strict regulatory aspects of labeling, information that is readable, useful, understandable, and in the patient’s own language should be placed on container labels in order to encourage the patients to read them, and in order to reduce medication errors [17–21]. As reported in other studies, pictograms on container labels could be useful [22]. In addition, sponsors should offer prescription forms designed for a specific clinical trial to ensure that at least the denomination of the drug is the same as on the container label. Written instructions for patients (documents designed by the sponsor specifically for the clinical trial) may also be useful when used in addition to oral instructions. However, drafting of these patient-directed materials requires taking into account the level of readability of these documents [23]. Future surveys could be conducted that include health literacy and patient adherence in the patient medication knowledge assessment.

Finally, due to the small sample size and the possibility of selection bias due to the recruitment of participants included in clinical trials undertaken in university hospitals, the results of this survey may not be applied other than to the study participants. We could not assess the possibility of a center-effect. Nevertheless, the COMQUEST survey identified major concerns regarding the medication knowledge of ambulatory adult volunteers included in clinical trials.

## Conclusion

The COMQUEST survey highlighted that only a quarter of the adult outpatients included in a clinical trial have a maximum possible understanding score regarding their investigational medicinal products. Age and the level of education were independent risk factors of inadequate medication understanding. These results should encourage trial sponsors and all of the other parties involved in clinical trials to promote education sessions and information interventions for volunteers included in any clinical trials in order to overcome the potential barriers due to poor medication knowledge. Our results demonstrate a need to improve materials such as prescriptions and investigational medication labels in a user-focused process. Initiatives to improve the patient experience in clinical trials need to consider these results, by prioritizing patient involvement. Sponsors need to learn more about the suitability of the clinical materials that are currently provided and obtain the patients’ opinions about their experiences and suggestions for improvements. Taking this into account could help increase patient understanding of complex information, allow patients to become better informed about their clinical trial medications, as well as improve protocol compliance to reduce risks.

## Supporting information

S1 File Survey questionnaire in French. (DOCX)

S2 File Survey questionnaire in English. (DOCX)

S3 File Database of the survey. (XLS)
