# Supplementary material for: What do adult outpatients included in clinical trials know about the investigational drugs being assessed: A cross-sectional study in France
Source: PLoS One. 2019 Aug 13;14(8):e0220383. doi: 10.1371/journal.pone.0220383 (PMC6692008; doi:10.1371/journal.pone.0220383)
Supplement: S1 File — (DOCX) [file pone.0220383.s001.docx]

**ENQUETE « COMQUEST » : QUESTIONNAIRE**

| Sexe :  DDN (MM/AAAA) :  Niveau d’étude^*^ (plus haut niveau atteint) : | Promoteur : Phase :  Code de l’étude :  Participant : □ volontaire sain □ patient  Nature de l’essai : □ ouvert □ simple aveugle □ double aveugle | Identification Pharmacie : |
| --- | --- | --- |

* cours élémentaire / collège (brevet des collèges) et CAP / lycée (bac) / bac +2 / bac +4 / bac +5 ou plus

| Critères d’inclusion (cocher)  □ participant > 18 ans  □ personne ambulatoire  □ première dispensation dans le cadre de l’étude dans laquelle le participant a été inclus  □ personne participant à un essai clinique dont les médicaments ne sont pas administrés à domicile par un professionnel de santé |
| --- |

| Prérequis (cocher)  □ les médicaments sont dispensés en accord avec le protocole et la copie de l’ordonnance est remise au participant  □ la personne est naïve de conseils pharmaceutiques avant l’entretien  □ la personne qui se prête à l’entretien est bien la personne à qui sont destinés les médicaments  □ la personne accepte de participer à l’enquête |
| --- |

Entretien individuel en tête-à-tête

| ***1 - « Avez-vous déjà participé à un essai clinique ? »***  □ Oui □ Non | | Med  1 | | Med  2 | | Med  3 | | Med  4 | | Med  5 | |
| --- | --- | --- | --- | --- | --- | --- | --- | --- | --- | --- | --- |
|  | | O | N | O | N | O | N | O | N | O | N |
| La personne connaît l’indication  ***2 - « Pouvez-me dire à quoi sert votre traitement ? »*** | | □ Oui □ Non | | | | | | | | | |
| La personne connaît le nom  **3 - « Pouvez-vous me citer le nom du/des médicaments que je vous remets ? »** | |  |  |  |  |  |  |  |  |  |  |
| La personne connaît la galénique  **4 - « Pouvez-vous me dire sous quelle forme se présente le/les médicaments ? »** | |  |  |  |  |  |  |  |  |  |  |
| La personne connaît la voie d’administration  ***5 - « Pouvez-vous me dire par quelle voie vous allez vous administrer le/les médicaments ? » (par exemple médicament à mettre sur la peau, à mettre sous la langue, à injecter, à avaler...)*** | |  |  |  |  |  |  |  |  |  |  |
| La personne connaît les modalités de prise | ***6 - « Combien de fois dans la journée allez-vous prendre votre traitement ? »*** |  |  |  |  |  |  |  |  |  |  |
|  | ***7 - « Combien d’unité de traitement (gélule, comprimé) allez-vous prendre à chaque fois ? »*** |  |  |  |  |  |  |  |  |  |  |
|  | ***8 - « Quelle est la durée/rythme/schéma de votre traitement avec ce que je vous remets ? »*** |  |  |  |  |  |  |  |  |  |  |
| La personne connaît les modalités de stockage  **9 - « Pouvez-vous me dire dans quelles conditions vous allez conserver le/les médicaments chez vous ? »** | |  |  |  |  |  |  |  |  |  |  |
| TOTAL (à remplir par le CHU de Nantes) | |  | |  | |  | |  | |  | |

Observations (enquêteur)

| Selon l’enquêteur, la personne parle-t-elle français ? | □ Oui □ Non | | |
| --- | --- | --- | --- |
| La personne a-t-elle ouvert la brochure (booklet) au cours de l’entretien ? | □ Oui □ Non □ NA | | |
| La personne cherche-t-elle des informations sur l’ordonnance ?  La personne cherche-t-elle des informations sur l’étiquette ?  La personne cherche-t-elle des informations sur un autre support ? | □ Oui □ Non  □ Oui □ Non  □ Oui □ Non Si oui, précisez : .................... | | |
| Sur quel support la personne cherche-t-elle majoritairement les informations ? | □ ordonnance □ étiquette □ NSP □ NA | | |
| Date : - - / - - / - - - - | | | Signature enquêteur – nom – fonction : |

**JOINDRE UNE COPIE ANONYMISÉE DE L’ORDONNANCE ET LES PHOTOS DES ÉTIQUETTES**
