# Supplementary material for: What do adult outpatients included in clinical trials know about the investigational drugs being assessed: A cross-sectional study in France
Source: PLoS One. 2019 Aug 13;14(8):e0220383. doi: 10.1371/journal.pone.0220383 (PMC6692008; doi:10.1371/journal.pone.0220383)
Supplement: S2 File — (DOCX) [file pone.0220383.s002.docx]

**“COMQUEST” SURVEY: QUESTIONNAIRE**

| Gender:  DOB (MM/YYYY):  Highest level of education^*^: | Sponsor: Study phase:  Study code:  Participant: □ healthy volunteer □ patient  Blinding: □ open label □ simple blinded □ double blinded | Pharmacy identification: |
| --- | --- | --- |

* No qualification; Initial professional diploma; High school degree; 2-year university degree; 4-year university degree; 5-year university degree or more

| Inclusion criteria  □ participant > 18 years  □ ambulatory participant included in any clinical trial  □ participant not been treated in this clinical trial previously  □ self-administered experimental drugs at home |
| --- |

| Requirements  □ experimental drugs dispensed in accordance with the protocol requirements and with a copy of the prescription form  □ no pharmaceutical advice before the interview  □ experimental drugs really dispensed to the patient or the healthy volunteer included in the clinical trial  □ the participant’s consent has been obtained |
| --- |

Face-to-face interview:

| ***1 -*** Previous participation in any clinical trial  □ Yes □ No | | Med  1 | | Med  2 | | Med  3 | | Med  4 | | Med  5 | |
| --- | --- | --- | --- | --- | --- | --- | --- | --- | --- | --- | --- |
|  | | Y | N | Y | N | Y | N | Y | N | Y | N |
| Therapeutic indication  ***2 – “Why do you have to take / use this medicine?”*** | | □ Yes □ No | | | | | | | | | |
| Name of the medicinal product  **3 – “What is the name of this medication?”** | |  |  |  |  |  |  |  |  |  |  |
| Pharmaceutical form  **4 - “What is the pharmaceutical form of this medication?”** | |  |  |  |  |  |  |  |  |  |  |
| Route of administration  ***5 – “How should you take / use this medication?”*** | |  |  |  |  |  |  |  |  |  |  |
| Frequency of intake, daily dose, and duration of treatment | ***6 - “How many times a day should you take / use this medication?”*** |  |  |  |  |  |  |  |  |  |  |
|  | ***7 – “How much should you take / use of this medication at each intake?”*** |  |  |  |  |  |  |  |  |  |  |
|  | ***8 – “How long do you have to take / use this medication?”*** |  |  |  |  |  |  |  |  |  |  |
| Storage conditions  **9 – “How should you keep your medication at home?”** | |  |  |  |  |  |  |  |  |  |  |
| TOTAL (to be completed by Nantes University Hospital) | |  | |  | |  | |  | |  | |

Observations by the interviewer:

| Does the participant speak French? | □ Yes □ No | | |
| --- | --- | --- | --- |
| Does the participant open the booklet during the interview? | □ Yes □ No □ Not applicable | | |
| Does the participant search information on the prescription form to answer?  Does the participant search information on the medication label or booklet to answer?  Does the participant search information on another specific document to answer? | □ Yes □ No  □ Yes □ No  □ Yes □ No If yes, specify: .................... | | |
| Which document does the participant mainly search for information on? | □ Prescription form □ Label or booklet □ I don’t know □ Not applicable | | |
| Date: - - / - - / - - - - | | | Interviewer’s signature – name and role: |

**PLEASE ATTACH AN ANONYMOUS COPY OF THE EXPERIMENTAL DRUGS PRESCRIPTION FORM AND**

**ATTACH PICTURES OF EACH EXPERIMENTAL DRUG LABEL**
